# Eyes Wide Shut: A Cohort Study Questioning the Role of Fundoscopy in Infective Endocarditis Diagnosis

**DOI:** 10.1093/cid/ciae067

**Published:** 2024-02-08

**Authors:** Elisavet Stavropoulou, Benoit Guery, Georgios Tzimas, Yan Guex-Crosier, Florence Hoogewoud, Piergiorgio Tozzi, Matthias Kirsch, Pierre Monney, Matthaios Papadimitriou-Olivgeris

**Affiliations:** Infectious Diseases Service, Lausanne University Hospital and University of Lausanne, Lausanne, Switzerland; Infectious Diseases Service, Lausanne University Hospital and University of Lausanne, Lausanne, Switzerland; Department of Cardiology, Lausanne University Hospital and University of Lausanne, Lausanne, Switzerland; Department of Ophthalmology, Jules-Gonin Eye Hospital and University of Lausanne, Lausanne, Switzerland; Department of Ophthalmology, Jules-Gonin Eye Hospital and University of Lausanne, Lausanne, Switzerland; Department of Cardiac Surgery, Lausanne University Hospital and University of Lausanne, Lausanne, Switzerland; Department of Cardiac Surgery, Lausanne University Hospital and University of Lausanne, Lausanne, Switzerland; Department of Cardiology, Lausanne University Hospital and University of Lausanne, Lausanne, Switzerland; Infectious Diseases Service, Lausanne University Hospital and University of Lausanne, Lausanne, Switzerland; Infectious Diseases Service, Cantonal Hospital of Sion and Institut Central des Hôpitaux, Sion, Switzerland

**Keywords:** infective endocarditis, Duke criteria, fundoscopy, Roth spots, ocular embolic events

## Abstract

In this retrospective/prospective study, we assessed the role of fundoscopy in 711 episodes with suspected infective endocarditis (IE); 238 (33%) had IE. Ocular embolic events (retinal emboli or chorioretinitis/endophthalmitis) and Roth spots were found in 37 (5%) and 34 (5%) episodes, respectively, but had no impact on IE diagnosis.

## Teaser

Fundoscopy among patients with suspected infective endocarditis detected ocular embolic events and Roth spots in 5% of episodes each but failed to substantially reclassify patients according to Duke criteria, suggesting that universal fundoscopy for diagnostic purposes is not justified.

## Graphical Abstract

**Graphical Abstract:**
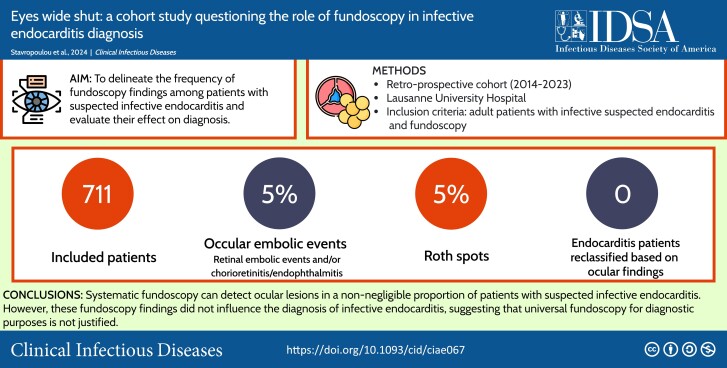
This graphical abstract is also available at Tidbit: https://tidbitapp.io/tidbits/eyes-wide-shut-a-cohort-study-questioning-the-role-of-fundoscopy-in-infective-endocarditis-diagnosis

Prompt identification of infective endocarditis (IE) and its associated complications is the key to improving outcomes. Fundoscopy is considered part of the initial workup of patients with suspected IE [1] since it can detect embolic events and Roth spots (2% of patients with definite IE) [2], which are 2 of the minor Duke criteria (vascular and immunological phenomena) to improve the diagnostic process [3].

While fundoscopy is currently considered a standard component of the workup for patients [1], there are limited data on fundoscopy findings [2, 4–6], and its impact on diagnosis has not been previously assessed. Therefore, our objective was to delineate the frequency of fundoscopy findings among patients with suspected IE and evaluate their effect on diagnosis.

## METHODS

This single-center study was conducted at the Lausanne University Hospital, Switzerland, from January 2014 to June 2023 (2014–2017, retrospective cohort; 2018 onward, prospective cohort). The Canton of Vaud Ethics Committee approved the study.

All adult patients (aged ≥18 years) with suspected IE (patients who had blood cultures drawn and an echocardiography performed specifically for the research of IE), fundoscopy performed and written consent (prospective cohort), or absence of refusal to use their data (retrospective cohort) were included. At our institution, fundoscopy by an ophthalmologist is part of the standard workup in patients with suspected IE. Episodes were classified as IE by the Endocarditis Team.

Demographic, clinical, microbiological, follow-up, and management data were collected. Using the 2023 Duke–International Society of Cardiovascular Infectious Diseases clinical criteria [3], we established 2 classifications based on clinical criteria: without and with the inclusion of fundoscopy findings. Using these 2 assessments, we calculated the reclassification rate (from rejected to possible and from possible to definite IE) for all patients with suspected IE and within the IE subgroups.

SPSS version 26.0 (SPSS, Chicago, IL) software was used for data analysis. Categorical variables were analyzed using the *χ* ^2^ or Fisher exact test, and continuous variables were analyzed using the Mann–Whitney *U* test. All statistical tests were 2-tailed, and *P* < .05 was considered statistically significant.

## RESULTS

Among the 1749 episodes with suspected IE, 711 (41%) underwent fundoscopy, among whom 238 (33%) were diagnosed with IE. For the remaining 473 episodes, the final diagnoses included other types of infections (402; 85%), autoimmune diseases (16; 4%), and other conditions (35; 12%).

Ocular embolic events (OEEs) were observed in 37 (5%) episodes, with 24 having retinal embolic events and 13 having chorioretinitis/endophthalmitis. Of these, 25 (11%) were among IE episodes and 12 (5%) without IE (Table 1). In 23 (64%) episodes, the Duke vascular phenomena criterion had been met before fundoscopy. Roth spots were found in 34 (5%) episodes, with 22 (9%) in IE episodes and 12 (5%) without IE. Among them, the Duke immunological phenomena criterion had been fulfilled in 1 (3%) episode prior to the fundoscopy. Fundoscopy results (OEEs and Roth spots) led to the reclassification of 11 (2%) episodes from rejected to possible IE and 2 (0.3%) from possible to definite IE. However, all of these reclassified cases did not have IE based on the Endocarditis Team’s evaluation.

**Table 1.** Comparison of Episodes With and Without Infective Endocarditis

Characteristics	No Infective Endocarditis (n = 473)		Infective Endocarditis (n = 238)		*P* Value
Demographics					
Male sex	303	64%	168	71%	.093
Age, y	67	54–78	68	55–77	.684
Charlson comorbidity index, points	4	2–7	5	2–7	.423
Cardiac predisposing factors					
Intravenous drug use	21	4%	23	10%	.008
Prosthetic valve	35	7%	63	27%	<.001
Prior endocarditis	15	3%	22	9%	.001
Cardiac implantable electronic devices	27	6%	57	24%	<.001
Minor predisposition criterion	117	25%	157	66%	<.001
Microbiological data					
Bacteremia/fungemia	341	72%	223	94%	<.001
*Staphylococcus aureus*	162	34%	102	43%	.027
Coagulase-negative staphylococci	27	6%	12	5%	.862
*Streptococcus* spp.	65	14%	61	26%	<.001
*Enterococcus* spp.	37	8%	31	13%	.031
Gram-positive other than staphylococci, streptococci, and enterococci	16	3%	5	2%	.482
HACEK	1	0.2%	4	2%	.045
Gram-negative other than HACEK	39	8%	7	3%	.006
Fungi	31	7%	6	3%	.021
Persistent bacteremia/candidemia (48 h)	69	15%	84	35%	<.001
Major microbiological criterion	214	45%	206	87%	<.001
Minor microbiological criterion	132	28%	22	9%	<.001
Imaging data					
Positive echocardiography (either TTE or TEE) for vegetation, perforation, abscess, aneurysm, pseudoaneurysm, fistula	10	2%	143	60%	<.001
Abnormal metabolic activity in ^18^F-fluorodeoxyglucose positron emission tomography/computed tomography	2	0.4%	38	16%	<.001
Positive cardiac computed tomography	1	0.2%	13	6%	<.001
Major imaging criterion	11	2%	172	72%	<.001
Manifestations					
Minor fever criterion	372	79%	199	84%	.134
Vascular phenomena before fundoscopy (major arterial emboli, septic pulmonary infarcts, mycotic aneurysm, intracranial hemorrhage, conjunctival hemorrhages, and Janeway's lesions)	65	14%	103	43%	<.001
Cerebral embolic events	30	6%	50	21%	<.001
Noncerebral embolic events	46	7%	76	32%	<.001
Ocular embolic events	12	3%	25	11%	<.001
Retinal embolic events	5	1%	19	8%	<.001
Chorioretinitis/endophthalmitis	7	2%	6	3%	.377
Minor vascular criterion before fundoscopy	65	14%	103	43%	<.001
Minor vascular criterion after fundoscopy	77	16%	108	45%	<.001
Immunological phenomena before fundoscopy: positive rheumatoid factor, glomerulonephritis, Osler nodes	11	2%	12	5%	.071
Positive rheumatoid factor	11	2%	6	3%	1.000
Glomerulonephritis	0	0%	6	3%	.001
Osler nodes	0	0%	3	1%	.037
Roth spots	12	3%	22	9%	<.001
Minor immunologic criterion before fundoscopy	11	2%	12	5%	.071
Minor immunologic criterion after fundoscopy	23	5%	33	14%	<.001
Data on surgery/CIED-extraction/histopathology					
Valve surgery performed	7	2%	74	31%	<.001
Major surgical criterion	0	0%	0	0%	…
CIED-extraction (among 84 patients with CIED)	0	0%	29	51%	<.001
Autopsy performed	4	0.8%	9	4%	0.013
Classification according to 2023 Duke–International Society of Cardiovascular Infectious Diseases clinical criteria					
Classification before fundoscopy					
Rejected	227	48%	7	3%	…
Possible	242	51%	61	26%	…
Definite	4	0.8%	170	71%	<.001
Classification after fundoscopy					
Rejected	214	45%	7	3%	…
Possible	253	54%	61	26%	…
Definite	6	2%	170	71%	<.001

Data are depicted as number/percentage or median/Q1–Q3.

Abbreviations: CIED, cardiac implantable electronic device; HACEK, *Haemophilus* spp., *Aggregatibacter* spp., *Cardiobacterium hominis*, *Eikenella corrodens*, *Kingella kingae*; TEE: transesophageal echocardiography; TTE: transthoracic echocardiography.

The comparison of episodes with suspected IE with and without OEEs is shown in Supplementary Table 1. OEEs were more common in episodes with fungemia (22% vs 4%; *P* < .001), persistent bacteremia/candidemia for 48 hours (49% vs 20%; *P* < .001), embolic events other than OEEs (54% vs 22%; *P* < .001), immunological phenomena (38% vs 6%; *P* < .001), and IE (68% vs 32%; *P* < .001).

The comparison of IE episodes with and without OEEs is presented in Supplementary Table 2. OEEs were more common in *Staphylococcus aureus* IE (72% vs 40%; *P* = .003), persistent bacteremia/candidemia for 48 hours (56% vs 33%; *P* = .027), vegetation ≥10 mm (56% vs 34%; *P* = .047), intracardiac abscess (40% vs 14%; *P* = .002), embolic events other than OEEs (80% vs 39%; *P* < .001), and immunological phenomena (44% vs 10%; *P* < .001).

## DISCUSSION

In our study, fundoscopy was performed in 41% of patients with suspected IE and revealed OEEs (either retinal emboli or chorioretinitis/endophthalmitis) and Roth spots in a small proportion of patients (in 5% of episodes each). Previous studies on IE patients reported similar rates of OEEs (8%–19%) [4, 5] but lower rates of Roth spots (2%) [2].

To the best of our knowledge, our study is the most comprehensive effort to explore the role of fundoscopy in diagnosing IE. However, the presence of OEEs and Roth spots had no impact on IE diagnosis, as the 13 patients reclassified according to Duke clinical criteria based on the fundoscopy findings did not have IE. Therefore, universal fundoscopy for diagnostic purposes in patients with suspected IE seems unnecessary [1]. The lack of impact on diagnosis can be attributed, in part, to the nonspecific nature of Roth spots. Although commonly linked with subacute IE, they can be found in a diverse array of noninfectious diseases, such as collagen vascular disease, leukemia, hypertensive or diabetic retinopathy, preeclampsia, and anoxia, underscoring their limited diagnostic specificity [7].

Among patients with suspected IE, OEEs were linked to fungemia, reinforcing the recommendation for universal fundoscopy screening in candidemia cases [8]. As expected, OEEs were more common among IE patients. In a large cohort of bloodstream infections and OEEs, IE was the second most common focus of infection (14%), following skin and soft tissue infections (17%) [9]. In the present study among *S. aureus* bacteremia episodes, OEEs were found in 7%, consistent with previous findings (9%) [6]. As expected, OEEs were associated with embolic events outside the eye and factors generally linked to systemic embolization, such as large vegetations (≥10 mm), *S. aureus*, persistent bacteremia/candidemia, and intracardiac abscess [10, 11].

This study has several limitations. First, it was a single-center study, and some patients were retrospectively included. Nevertheless, it is the most extensive study to date on fundoscopy's impact on patients with suspected IE. Second, despite being part of the standard diagnostic workup, fundoscopy was performed in fewer than half of the patients with suspected IE. This can be explained by the fact that the decision to perform fundoscopy remained at the discretion of the physician.

In conclusion, this study has confirmed that systematic fundoscopy can detect ocular lesions in a nonnegligible proportion of patients with suspected IE. However, these fundoscopy findings did not influence the diagnosis of IE, suggesting that universal fundoscopy for diagnostic purposes is not justified. Nevertheless, in cases where patients exhibit ocular symptoms or have fungemia, fundoscopy is warranted.

## Supplementary Data

Supplementary materials are available at *Clinical Infectious Diseases* online. Consisting of data provided by the authors to benefit the reader, the posted materials are not copyedited and are the sole responsibility of the authors, so questions or comments should be addressed to the corresponding author.

## Supplementary Material

ciae067_Supplementary_Data

## Notes

***Author Contributions.*** M. P. O., P. M., and B. G. conceived the idea. E. S., B. G., G. T., Y. G. C., F. H., P. T., M. K., P. M., and M. P. O. collected the patient data. M. P. O. supervised the project. M. P. O. performed the analysis and interpreted the results. E. S. wrote the manuscript. All authors contributed to manuscript revision and read and approved the submitted version.

## References

[1] Cahill TJ, Prendergast BD. Infective endocarditis. Lancet 2016; 387:882–93. 26341945 10.1016/S0140-6736(15)00067-7

[2] Murdoch DR, Corey GR, Hoen B, et al Clinical presentation, etiology, and outcome of infective endocarditis in the 21st century: the International Collaboration on Endocarditis—prospective cohort study. Arch Intern Med 2009; 169:463–73. 19273776 10.1001/archinternmed.2008.603 PMC3625651

[3] Fowler VG, Durack DT, Selton-Suty C, et al The 2023 Duke-ISCVID criteria for infective endocarditis: updating the modified Duke criteria. Clin Infect Dis 2023; 77:518–26.37138445

[4] Bergmans T, De Meester P, Herregods MC. Impact of nuclear imaging on diagnosis and management of infective endocarditis. Acta Cardiol 2020; 75:348–52. 30982414 10.1080/00015385.2019.1595268

[5] Van Riet J, Hill EE, Gheysens O, et al (18)F-FDG PET/CT for early detection of embolism and metastatic infection in patients with infective endocarditis. Eur J Nucl Med Mol Imaging 2010; 37:1189–97. 20204357 10.1007/s00259-010-1380-x

[6] Jung J, Lee J, Yu SN, et al Incidence and risk factors of ocular infection caused by *Staphylococcus aureus* bacteremia. Antimicrob Agents Chemother 2016; 60:2012–7. 26824952 10.1128/AAC.02651-15 PMC4808144

[7] Ruddy SM, Bergstrom R, Tivakaran VS. Roth Spots. StatPearls. Treasure Island (FL): StatPearls Publishing, 2024. 29494053

[8] Pappas PG, Kauffman CA, Andes DR, et al Clinical practice guideline for the management of candidiasis: 2016 update by the Infectious Diseases Society of America. Clin Infect Dis 2016; 62:e1–50. 26679628 10.1093/cid/civ933 PMC4725385

[9] Vaziri K, Pershing S, Albini TA, Moshfeghi DM, Moshfeghi AA. Risk factors predictive of endogenous endophthalmitis among hospitalized patients with hematogenous infections in the United States. Am J Ophthalmol 2015; 159:498–504. 25486541 10.1016/j.ajo.2014.11.032

[10] Papadimitriou-Olivgeris M, Guery B, Ianculescu N, et al Risk of embolic events before and after antibiotic treatment initiation among patients with left-side infective endocarditis. Infection 2024; 52:117–28. 37402113 10.1007/s15010-023-02066-z PMC10811187

[11] Yang A, Tan C, Daneman N, et al Clinical and echocardiographic predictors of embolism in infective endocarditis: systematic review and meta-analysis. Clin Microbiol Infect 2019; 25:178–87. 30145401 10.1016/j.cmi.2018.08.010

