# Supplementary material for: Eyes Wide Shut: A Cohort Study Questioning the Role of Fundoscopy in Infective Endocarditis Diagnosis
Source: Clin Infect Dis. 2024 Feb 8;78(3):663–6. doi: 10.1093/cid/ciae067 (PMC10954338; doi:10.1093/cid/ciae067)
Supplement: ciae067_Supplementary_Data [file ciae067_supplementary_data.pdf]

Supplementary Table 1. Predictors of ocular embolic events among patients with suspected infected endocarditis

	**Without ocular embolic events (n=674)**		**With ocular embolic events (n=37)**		***P***
Demographics					
Male sex	447	66%	24	65%	0.860
Age (years)	68	54-78	69	55-77	0.911
Charlson Comorbidity Index (points)	5	2-7	5	2-8	0.697
Cardiac predisposing factors (minor predisposition criterion)	263	39%	11	30%	0.300
Microbiological data					
Bacteremia/fungemia	531	79%	33	89%	0.148
S. aureus	246	37%	18	49%	0.162
Coagulase-negative staphylococci	39	6%	0	0%	0.255
Streptococcus spp	123	18%	3	8%	0.181
Enterococcus spp	66	10%	2	5%	0.567
Gram-positive other than staphylococci, streptococci and enterococci)	21	3%	0	0%	0.620
HACEK	5	0.7%	0	0%	1.000
Gram-negative other than HACEK	44	7%	2	5%	1.000
Fungi	29	4%	8	22%	<0.001

Persistent bacteremia/candidemia (48h)	135	20%	18	49%	<0.001
Major imaging criterion	161	24%	22	60%	<0.001
Manifestations					
Minor fever criterion	538	80%	33	89%	0.204
Vascular phenomena other than ocular	148	22%	20	54%	<0.001
Cerebral embolic events	69	10%	11	30%	0.001
Non-cerebral embolic events	94	14%	18	49%	<0.001
Immunological phenomena	42	6%	14	38%	<0.001
Positive rheumatoid factor	15	2%	2	5%	0.220
Glomerulonephritis	6	0.9%	0	0%	1.000
Osler nodes	2	0.3%	1	3%	0.148
Roth spots	22	3%	12	32%	<0.001
Infective endocarditis	213	32%	25	68%	<0.001

Data are depicted as number/percentage or median/Q1-Q3

HACEK: *Haemophilus* spp, *Aggregatibacter* spp, *Cardiobacterium hominis*, *Eikenella corrodens*, *Kingella kingae*

Supplementary Table 2. Predictors of ocular embolic events among patients with infected endocarditis

	**Without ocular embolic events (n=213)**		**With ocular embolic events (n=25)**		***P***
Demographics					
Male sex	152	71%	16	64%	0.488
Age (years)	68	54-77	70	55-76	0.911
Charlson Comorbidity Index (points)	5	2-7	3	2-7	0.697
Cardiac predisposing factors (minor predisposition criterion)	146	69%	11	44%	0.024
Microbiological data					
S. aureus	85	40%	18	72%	0.003
Coagulase-negative staphylococci	12	6%	0	0%	0.621
Streptococcus spp	59	28%	3	12%	0.146
Enterococcus spp	29	14%	2	8%	0.752
Gram-positive other than staphylococci, streptococci and enterococci)	7	3%	0	0%	1.000
HACEK	4	2%	0	0%	1.000
Gram-negative other than HACEK	6	3%	1	4%	0.545
Fungi	5	2%	1	4%	0.490
Persistent bacteremia/candidemia (48h)	70	33%	14	56%	0.027

Intracellular bacteria	4	2%	0	0%	1.000
Major imaging criterion	150	70%	22	88%	0.096
Vegetation	142	67%	19	76%	0.498
Vegetation ≥10mm	73	34%	14	56%	0.047
Abscess	29	14%	10	40%	0.002
Other lesions*	33	16%	7	28%	0.152
Site of infection					
Aortic valve	81	38%	10	40%	0.832
Mitral valve	68	32%	10	40%	0.500
Tricuspid valve	15	7%	2	8%	0.695
Pulmonary valve	2	0.9%	0	0%	1.000
CIED-lead infective endocarditis	16	8%	2	8%	1.000
Type of left-side valve					
Native	107	50%	17	68%	0.137
Prosthetic	43	20%	4	16%	0.793
Manifestations					
Minor fever criterion	176	83%	23	92%	0.389
Vascular phenomena other than ocular	83	39%	20	80%	<0.001
Cerebral embolic events	39	18%	11	44%	0.007

Non-cerebral embolic events	58	27%	18	72%	<0.001
Immunological phenomena	22	10%	11	44%	<0.001
Positive rheumatoid factor	5	2%	2	8%	0.122
Glomerulonephritis	6	3%	0	0%	1.000
Osler nodes	2	0.9%	1	4%	0.284
Roth spots	13	6%	9	36%	<0.001

Data are depicted as number/percentage or median/Q1-Q3

CIED: cardiac implantable electronic devices; HACEK: *Haemophilus* spp, *Aggregatibacter* spp, *Cardiobacterium hominis*, *Eikenella corrodens*, *Kingella kingae*

*Perforation, dehiscence of prosthetic valve, fistula, pseudoaneurysm, aneurysm
